# Association between stage at diagnosis and knowledge on cervical cancer among patients in a Kenyan tertiary hospital: a cross-sectional study

**DOI:** 10.11604/pamj.supp.2016.25.2.10684

**Published:** 2016-11-26

**Authors:** Kabura Wamburu, Naftali Busakhala, Kevin Owuor, Josephat Nyagero

**Affiliations:** 1 Moi University, School of Public Health, Nairobi, Kenya; 2 AMREF Health Africa, Kenya; 3 United States Agency for International Development, Nairobi, Kenya

**Keywords:** Human papillomavirus, cervical cancer screening, symptoms

## Abstract

**Introduction:**

In Kenya, cervical cancer is the second most common cancer among women; almost half of all women with invasive cervical cancer are diagnosed at a late stage. Few women are aware of the symptoms and risk factors of cervical cancer and that its precursor lesions are detectable through screening thus most women seek treatment when the cancer is at an advanced stage. The study explored the influence of cervical cancer awareness on stage at diagnosis in patients attending Kenyatta National Hospital.

**Methods:**

A cross-sectional survey was adapted to obtain socio-demographic information, knowledge on symptoms and risk factors from 361 women with histological diagnosis of cervical cancer conveniently sampled at Kenyatta National Hospital. Associations between stage at diagnosis and knowledge on cervical cancer were tested using chi-square statistic and fisher's exact test at 95% confidence interval.

**Results:**

Seven in every 10 women (72.6%) presented with advanced stage cervical cancer. Knowledge on the sexually transmitted nature of cervical cancer was inadequate, 22% of women with early stage and 23.7% of women with advanced stage cervical cancer (p=0.874). Majority of the women were not aware of the causative link between cervical cancer and human papillomavirus (HPV), 8 (13.1%) of women with early stage and 5 (3.5%) of women with advanced stage cervical cancer (p=0.036).

**Conclusion:**

Stage at presentation was advanced and knowledge on the role of a sexually transmitted virus in the cervical cancer aetiology was poor among the women. Increasing screening programs and providing information highlighting this association is necessary.

## Introduction

Cervical cancer is the third most frequently diagnosed cancer and the fourth leading cause of cancer deaths among women with approximately 529,800 new cancer cases and 275,100 cancer deaths occurring in women worldwide. Close to 86% of these new cases and deaths occur in developing countries [1]. Sub-Saharan Africa has a disproportionately enormous burden of cervical cancer which is mostly due to scarce screening programs that allow for early detection of precancerous lesions and early stage cervical cancer [2]. In Kenya, cervical cancer is the second most prevalent cancer after breast cancer [3]. The World Health Organisation (WHO) estimates that approximately 4802 women were diagnosed with cervical cancer in 2012 with about 2451 of the incident cases dying of the disease [4]. The major underlying cause of cervical cancer is human papillomavirus (HPV) infection and its precursor lesions [5]. Smoking has been found to be an independent risk factor for cervical cancer after altering the effects of HPV infection [6]. Other risk factors for cervical cancer include having many sexual partners, high parity, early age at first intercourse, co-infection with human immunodeficiency virus (HIV) and long term use of oral contraceptives [7]. Early detection of cancer is vital due to the documented relationship between stage at diagnosis and survival. Prevention amenities such as information on cervical cancer, screening services, vaccination against HPV, the causes of and treatment of pre-cancerous lesions are all vital in treating cervical cancer at its early stage [8]. Abridging cancer time of diagnosis is dependent on a patient presenting to a healthcare facility with probable cancer symptoms commonly referred to as patient delay, on primary healthcare providers reacting aptly to the symptoms, by either setting up additional investigations and or referring them to a specialist also known as doctor or practitioner delay and by minimising the interval between referral and diagnosis, referred to as hospital or system delay. However, patient delay is known to play a major role in most delays [9, 10]. Low levels of education make it difficult for the patients to understand the implications of the disease and to take note of the common symptoms. In African countries, approximately 95% of cancer patients are diagnosed with late stage or end stage disease. Culture, low level of cancer knowledge in the population, lack of specialized health care practitioners and limited access to health care facilities contribute to the delay in diagnosis for cancer patients [11, 12]. Similarly, poor implementation of cervical cancer screening programs is a likely cause of few women being screened in health facilities with the basic infrastructure and facilities for cervical cancer screening in the east, central and southern African countries (ECSA) [13]. Women at risk of developing cervical cancer require accurate information for them to understand prevention methods and to prompt them to use screening services. Although a few women might have knowledge on the disease, fear of the procedure, embarrassment concerning pelvic examination, family pressures, or fear of cancer may prevent them from seeking healthcare services [14]. This study aimed to establish the association between stage at diagnosis and knowledge on cervical cancer among women attending Kenyatta National Hospital (KNH).

## Methods

**Study design and study population:** a cross-sectional study was carried out on patients with cervical cancer attending KNH, a national referral hospital and primary teaching hospital for the school of medicine, University of Nairobi (UoN). This study focused on women above the age of 18 years with a histological diagnosis of cancer receiving treatment at the cancer treatment centre (CTC), radiotherapy department and the obstetrics and gynaecology department. Patients who were critically unwell thus unable to respond to questions, those in documented remission of cancer and those who were unwilling to take part in the study were excluded. The stage at presentation of cervical cancer based on histological diagnosis was abstracted from clinical records of patients. A total of 385 women attending the clinics were selected through convenience sampling after written informed consent was obtained during the period of May to July 2015; 24 women were excluded from the study as their clinical records did not have staging information. Overall, 361 out of the 385 eligible women were recruited.

**Data collection :** a semi-structured questionnaire was administered with the help of a trained research assistant who conducted face-to-face interviews. The questionnaire contained detailed questions concerning socio-demographic information such as age, marital status, age at marriage, parity, education level and partners' education level, occupation and total household income. Knowledge on cervical cancer risk factors; HPV, smoking and contraceptive use and symptoms such as vaginal bleeding between periods, bleeding after sex, vaginal discharge and bleeding after menopause were assessed. The study was approved by the Kenyatta National Hospital/University of Nairobi ethics and review committee (KNH/UON-ERC) as well as by the Institutional Research and Ethics Committee at Moi University College of Health Sciences (MUCHS-IREC).

**Data analysis:** the main outcome variable, stage at diagnosis, was determined using the International Federation of Gynaecology and Obstetrics (FIGO) staging system. For these analyses, stage at presentation was grouped as early (IA1, IA2, IB1 and IIA) or advanced (IIB, IIIA, IIIB, IVA and IVB). Stage at presentation was noted as the stage that a clinician reported at first diagnosis of malignancy. Data analysis was performed using the Statistical Analysis System, ver. 9 (SAS Inc., North Carolina, USA) and statistical significance was set at p ≤ 0.05. Statistical associations between stage at diagnosis and knowledge on cervical cancer were tested using chi-square statistic and fisher's exact test at 95% confidence interval.

## Results

The socio-demographic characteristics of the population (n=361) are shown in Table 1.

**Table 1:** Socio-demographic characteristics of participants

Variable	No. of cases (n=361)	%
**Age in years**		
≤40	77	21.4
41-50	123	34.2
51-60	106	29.4
≥61	54	15
**Age at marriage**		
≤15yrs	21	6.7
16-20yrs	150	48.4
21-25 yrs	109	34.7
≥26yrs	32	10.2
**Marital status**		
Single	48	13.4
Married	221	61.7
Divorced	25	7
Widowed	64	17.9
Parity		
0-3	146	40.6
4-7	175	48.6
≥8	39	10.8
**Education level**		
No formal education	37	10.3
Primary education	198	55
Secondary education	112	31.1
Tertiary education	11	3.1
University education	2	0.6
**Partner’s education level**		
No formal education	10	4.5
Primary education	93	42.1
Secondary education	103	46.6
Tertiary education	14	6.3
University education	1	0.5
**Occupation**		
Permanently employed	20	5.5
Casually employed (hairdresser/ house girl)	35	9.7
Unemployed (house wife/student)	69	19.1
Self-employed (farmer/business)	237	65.7
**Stage at diagnosis**		
Early	99	27.4
Late	262	72.6

The mean age of patients was 49 years (range, 42-57 years) and the mean age at marriage was 20 years (range, 18-23 years). Majority of the women were multiparous with 48.6% having between 4 and 7 children. The number of women diagnosed with advanced stage cervical cancer was higher than those diagnosed at an early stage (72.6% vs 27.4).

Table 2 summarises data on cervical cancer awareness and the relationship with stage at presentation. Knowledge on cervical cancer was low in women diagnosed with early and advanced stage cancer however, these differences were not statistically significant (62.6% vs 55.6%, respectively, p=0.226); the source of knowledge on cervical cancer was mostly through health education but with no statistical significance (p=0.163). Human papillomavirus was only mentioned as the cause of cervical cancer by 13.1% of women diagnosed with early stage and 3.5% of women diagnosed with advanced stage cancer (p=0.036). Most women diagnosed with early and advanced stage cervical cancer considered symptoms such as abnormal vaginal bleeding, bleeding after sex and post menopausal bleeding as a sign of infection and not cancer but the differences were not statistically significant. Similarly, vaginal discharge was attributed to infection by most women (p=0.04). There was no association between smoking and stage at presentation (p=0.526) as shown in Table 3.

**Table 2:** Differences in Knowledge on risk factors for cervical cancer and stage at presentation

	Early Stage		Advanced Stage		
	Number (n=99)	%	Number (n=262)	%	p value
**Have you ever heard of cervix cancer?** **(χ^2^ (1 df) = 1.468) ǂ**					0.226
Yes	62	62.6	145	55.6	
No	37	37.4	116	44.4	
Causes of cervical cancer †					**0.036**
Human immunodeficiency virus (HIV)	1	1.6	2	1.4	
Human papillomavirus (or genital warts)	8	13.1	5	3.5	
I don’t know	52	85.2	136	95.1	
**How did you learn about cervical cancer?+**					0.163
From neighbours	15	24.6	43	29.7	
Through the radio	28	45.9	62	42.8	
Through the television	11	18	13	9	
From the newspaper	3	4.9	1	0.7	
Through health education	32	52.5	87	60	
**Causes of abnormal vaginal bleeding ǂ**					0.625
Infection	42	42.4	93	35.8	
Cancer	13	13.1	35	13.5	
Other	43	43.4	127	48.8	
**What causes bleeding after sex? ǂ**					0.537
Infection	48	49	107	40.8	
Cancer	11	11.2	34	13	
Injury	8	8.2	16	6.1	
Other	30	30.6	103	39.3	
**What causes vaginal discharge? ǂ**					**0.04**
Infection	67	67.7	143	54.6	
Cancer	7	7.1	32	12.2	
Other	23	23.2	80	30.5	
**What causes bleeding after menopause? ǂ**					0.166
Infection	58	59.2	134	51.9	
Cancer	16	16.3	42	16.3	
Other	23	26.5	80	31	

ǂ Numbers may not add up to total because of missing data.

+ Indicates a multiple response question numbers may not add up to total.

† Indicates skipping patterns in the questionnaire, numbers may not add up to total.

**Table 3:** Knowledge on infectious and non-infectious risk factors and stage at presentation

	Early Stage		Advanced Stage		
	Number (n=99)	%	Number (n=262)	%	p Value
**Have you ever had genital warts? ǂ**					**0.038**
Yes	23	23.2	37	14.2	
No	76	76.8	217	83.1	
I don’t know	0	0	7	2.7	
**Tobacco use †**					0.526
Never	23	100	35	94.6	
Smoker	0	0	1	2.7	
Ex-smoker	0	0	1	2.7	
**Regular gynaecological examinations ǂ**					**0.001**
Yes	28	28.6	34	13	
No	70	71.4	227	87	
**What do gynaecological examinations help with? ǂ**					0.137
Early detection of carcinoma of the cervix	68	69.4	163	62.2	
To detect STIs	14	14.3	27	10.3	
Others	16	16.3	70	26.7	
**What is a pap smear test? ǂ**					**0.024**
It is a smear from the cervix used to rule out the presence of cancer cells	73	75.3	155	59.8	
I don’t know	24	24.7	103	39.8	
**Is cervical cancer sexually transmitted? ǂ**					0.874
Yes	22	22.4	62	23.7	
No	30	30.6	73	27.9	
I don’t know	46	46.9	127	48.5	
**Is promiscuity a risk factor ǂ**					0.216
Yes	46	46.5	116	44.3	
No	14	14.1	58	22.1	
I don’t know	39	39.4	88	33.6	
**Hormonal contraceptives use ǂ**					0.953
Yes	72	72.7	189	72.4	
No	27	27.3	72	27.6	
**Duration of contraceptive use categories †**					0.159
≤1yr	8	11.1	23	12.2	
2-8yrs	27	37.5	72	38.1	
9-15 yrs	22	30.6	74	39.2	
≥16yrs	15	20.8	20	10.6	

ǂ Numbers may not add up to total because of missing data

^+^Indicates a multiple response question numbers may not add up to total

† Indicates skipping patterns in the questionnaire, numbers may not add up to total

More than 70% of the women cited that they had never had genital warts (76.8% vs 83.1%, p=0.038). Women diagnosed with early and advanced stage disease did not frequently obtain gynaecological examinations (28.6% vs 13%, p=0.001). Awareness of the importance of obtaining regular gynaecological examinations was higher in women diagnosed with early stage cancer however with no statistical significance (69.4% vs 62.2%, p=0.137). Majority of the women diagnosed with early and advanced stage cancer cited that they did not know that cervical cancer could be sexually transmitted however, with no statistical significance (46.9% vs 48.5%, p=0.874). Hormonal contraceptive use was common in about 72% of the women diagnosed with early and advanced stage cancer but with no statistical significance (p=0.953).

## Discussion

This study demonstrated that majority of women presented with advanced stage cervical cancer. The most notable finding was the particularly low level of awareness of HPV as a causative agent of cervical cancer. This is consistent with other studies which found that about 40% of women had heard of HPV, but not more than half were aware that it caused cervical cancer [15]. In this study, awareness of HPV was linked to patients having had genital warts which would prompt them to seek treatment thereby enabling them to obtain information on HPV. Their ability to cite HPV as a cause of cervical cancer was also used as an indicator for awareness. Women's perception of cervical cancer would change substantially if HPV testing was introduced into cervical cancer screening programs as this would lessen the confusion in women who have had no previous knowledge of HPV or its connection to cervical cancer [16]. It was also observed that the number of women who were aware of cervical cancer prior to their diagnosis was low, consistent with studies in Nigeria that show low levels of awareness of cervical cancer [17]. There was also evidence that only a small proportion of the women were able to attribute cancer as a cause of most of the symptoms mentioned. This is consistent with other findings that showed that women assumed their symptoms were as a result of a continuation of their menses, genital infections and irregular menses [18]. Majority of the women correctly cited the need for frequent gynaecological examinations however, most were not screened regularly. This is consistent with findings that show that, in many developing countries, screening is opportunistic and is mostly characterised by poor coverage and lack of quality control systems [18]. Similarly, perceived barriers to screening such as cost, lack of information on where to obtain screening services and fear associated with pain from a Pap smear test hinder most women from being screened [14]. It was also observed that most of the women were not aware of the sexually transmitted nature of cervical cancer though some considered promiscuity to be a risk factor. This low level of awareness is consistent with other studies that show that information about the association between cervical cancer and sexual transmission or sexual activity is not well established [5]. Information regarding the link between cervical cancer and sexual activity is necessary in order to allow women to make informed choices concerning their sexual behaviour. However, care must be taken when providing this information to the public as the fear and stigma of associating cervical cancer with a sexually transmitted disease may deter women from taking up screening services [19].

**Strengths and limitations of the study:** one of the main strengths of this study is the use of information on the women's initial histological diagnosis which was obtained from the patient files. This enabled us to determine the proportion of patients who presented with early and advanced stage cervical cancer. Additionally, staging data for 94% of the participants was available. A further advantage of this study was the high response rate which reduced the likelihood of non-response bias. This study is subject to the limitation of self report data. However, the bias in the data was negligible because behavioural factors were not mainly being assessed. Studies have demonstrated that information on the frequency of cervical cancer screening in women is mostly based on their own account. The tendency to overestimate or underestimate their participation has been observed owing to the discordance between medical records and the patient's account of the number of Pap smears they have previously had [20]. Similarly, women who had had a gynaecological examination besides a Pap smear erroneously reported having had a Pap smear more frequently than women who hardly ever had gynaecological examinations [21].

## Conclusion

This study revealed that stage at presentation among women attending Kenyatta national hospital was considerably advanced. Knowledge on cervical cancer symptoms, on the link between HPV and cervical cancer and on the sexually transmitted nature of cervical cancer was limited. There is therefore need to increase screening programs and health education programs that will highlight these linkages.

### What is known about this topic

- Low levels of education, lack of knowledge and poverty are associated with inadequate preventive cervical cancer screening practices. Cervical cancer can be prevented if precancerous lesions are detected early through screening. Screening for cervical cancer only takes place in fragmented projects due to poorly revised national policy guidelines, scarce alternatives for diagnosis and treatment at secondary levels of care and ineffective monitoring and evaluation systems for ongoing projects;
- Lack of adequate knowledge on cervical cancer is not only limited to patients, healthcare workers may also be ill informed about the disease resulting in missed screening opportunities and delays in patient referrals to tertiary institutions;
- Infection with HPV is essential for the development of cervical cancer and is transmitted mainly through sexual contact. Infection with HPV is greatly increased in individuals who have multiple sexual partners, those who had early sexual debut and those who are co-infected with other sexually transmitted diseases such as herpes simplex virus, Chlamydia trachomatis. Vaccination against HPV provides immunity against about 70% of the strains that cause cervical cancer. This vaccine however is not available as part of the national vaccine and immunization program.

### What this study adds

- This study shows that the level of awareness of the causal link between HPV and cervical cancer in women presenting with early and advanced stage cancer is low;
- The study also demonstrates that women who were diagnosed with early stage disease also had inadequate knowledge of the rudimentary symptoms of cervical cancer and that public knowledge regarding the disease was low in these women;
- The study further demonstrated that only a small percentage of the women were aware of the sexually transmitted nature of cervical cancer.
